# Fbxw7 Tumor Suppressor: A Vital Regulator Contributes to Human Tumorigenesis

**DOI:** 10.1097/MD.0000000000002496

**Published:** 2016-02-18

**Authors:** Jun Cao, Ming-Hua Ge, Zhi-Qiang Ling

**Affiliations:** From the Zhejiang Cancer Research Institute (JC, Z-QL); and Department of Surgical Oncology, Zhejiang Province Cancer Hospital, Zhejiang Cancer Center, Hangzhou, China (JC, M-HG).

## Abstract

Rapidly accumulating data indicate that F-box/WD repeat-containing protein 7 (Fbxw7) is one of the most frequently mutated genes in human cancers and regulates a network of crucial oncoproteins. These studies have generated important new insights into tumorigenesis and may soon enable therapies targeting the Fbxw7 pathway.

We searched PubMed, Embase, and ISI Web of Science databases (1973–2015, especially recent 5 years) for articles published in the English language using the key words “Fbxw7,” “Fbw7,” “hCDC4,” and “Sel-10,” and we reviewed recent developments in the search for Fbxw7.

Fbxw7 coordinates the ubiquitin-dependent proteolysis of several critical cellular regulators, thereby controlling essential processes, such as cell cycle, differentiation, and apoptosis. Fbxw7 contains 3 isoforms (Fbxw7α, Fbxw7β, and Fbxw7γ), and they are differently regulated in subtract recognition. Besides those, Fbxw7 activity is controlled at different levels, resulting in specific and tunable regulation of the abundance and activity of its substrates in a variety of human solid tumor types, including glioma malignancy, nasopharyngeal carcinoma, osteosarcoma, melanoma as well as colorectal, lung, breast, gastric, liver, pancreatic, renal, prostate, endometrial, and esophageal cancers.

Fbxw7 is strongly associated with tumorigenesis, and the mechanisms and consequences of Fbxw7 deregulation in cancers may soon enable the development of novel therapeutic approaches.

## INTRODUCTION

Ubiquitin proteasome system can regulate many important cellular processes by degradating short-term proteins. The target proteins are ubiquitinated and degraded through a series of actions of ubiquitin ligase. The SKP1-CUL1-F-box (SCF) ubiquitin ligases mainly comprised of F-box protein, Skp1, Cullin 1, and Roc1/Rbx1/Hrt1. F-box/WD repeat-containing protein 7 (Fbxw7) is a member of the F-box protein family, which function as the substrate recognition component of the SCF E3 ubiquitin ligase. The Fbxw7 (also known as hCDC4, Sel-10) was first identified in 1973 by Hartwell^1^ at Washington University in his research for genetic control of the cell division cycle in yeast. Since this discovery in yeast, a wealth of experimental evidence, suggests that Fbxw7 is a tumor suppressor through negative regulation of many oncogenic proteins. It is also the substrate-recognition component of the SCF ubiquitin ligases that can bring together protein substrates and the catalytic core of the ubiquitin machinery. However, compared with the research of its substrates, little is known about the regulation of Fbxw7 itself, and how different Fbxw7 isoforms play in substrate recognition and ubiquitylation. Therefore, in the following sections, we will mainly discuss the Fbxw7 isoforms, the regulation of Fbxw7, and its role in different human solid tumors and its mechanisms for inactivation.

## ETHICAL REVIEW

The study presented here is a literature review and it does not need any ethical approval. This study does not involve interaction with any human subjects and does not collect any identifiable private information.

## METHODS

Relevant literature focusing on the field of Fbxw7 in human tumorigenesis was identified through searching in PubMed, Embase, and ISI Web of Science databases by keywords “Fbxw7,” “Fbw7,” “hCDC4,” and “Sel-10”, from 1973 to 2015, especially recent 5 years. There were no limitations imposed on language and study types. The references cited by the articles searched were also analyzed.

Three independent investigators (contributing authors), CJ, GMH, and LZQ, conducted the searching process and the writing manuscript. Relevant literature was chosen according to the objective of this review and the availability of full text.

### Fbxw7 Structure and Isoforms

The Fbxw7 gene locus maps to chromosome region 4q32, which is frequently deleted in a broad spectrum of human tumor types, and is composed of 4 untranslated and 13 coding exons spanning approximately 210 kb of the human genome. The Fbxw7 structure consists of a WD40-repeat domain, an F-box domain, a 5 residue tail, and an α-helical linker domain. The WD40 domain forms a canonical 8-bladed β-propeller structure, which consists of 4 antiparallel β strands and resembles a cylinder with narrow and wide ends and a solvent-filled central channel, and it can be combined with the specific substrate.

Mammalian cells contain 3 Fbxw7 isoforms (Fbxw7α, Fbxw7β, and Fbxw7γ), and they are derived from the same locus on chromosome 4. All isoforms contain a D domain, an F box domain, and a WD40-repeat domain. They share 10 C-terminal exons, which encode for the F-box and substrate-recognition motifs, and then direct a variety of substrates to degradation. Each isoform can identify substrate that contains a phosphorylated consensus Fbxw7 phospho-degron motif. However, other factors affect the interactions between a substrate and a specific isoform: the subcellular localization and the amount of Fbxw7 isoform at a specific setting. And their subcellular localization is determined by a unique N-terminal exon specific for each isoform. Fbxw7α, the most abundant isoform in proliferating cells, mainly localizes to the nucleoplasm, whereas Fbxwβ localizes to cytoplasm and Fbxw7γ localizes to nucleolus. Fbxw7α is thought to perform most Fbxw7 function. Yang et al^2^ reported that in breast cancers, the association of phosphatase and tensin homolog gene with both amplified in breast cancer 1 (AIB1) and Fbxw7α could lead to the downregulation of AIB1 transcriptional activity, which resulted in regulating the oncogenic function of AIB1. Furthermore, Fbxw7α regulated positively epidermal growth factor receptor (EGFR) by influencing a proteasome-dependent ubiquitination step essential for constitutive degradation and stability of EGFR. Strohmaier et al^3^ found that Fbxw7α was the predominant form in most human tissues including cancers, while Fbxw7β was mostly expressed as the form only in nonproliferating tissues like brain and skeletal muscle, and tended to be repressed in some cancers. These observations suggest that Fbw7α may control of the cell cycle in proliferating cells, and Fbxw7β may be needed for the permanent cell-cycle arrest that leads to senescence. What is more, van Drogen et al^4^ indicated that the combined activity of Fbxw7α and Fbxw7γ were essential for the degradation of cyclin E. In the study of Sionov et al,^5^ they showed that Fbxw7β was induced by all the stress stimuli tested, including vinblastine, cisplatinum, and etoposide treatments, in a p53-dependent manner. But the expression of Fbxw7α and Fbxw7γ was p53-independent and their response to most stress stimuli was limited. What is more, under certain conditions, the same genotoxic agent stimulated induction of Fbxw7β and repression of Fbxw7α. The expression of 3 isoforms is controlled by different promoters and upstream regulation elements, but the regulation mechanisms controlling the expression of 3 isoforms are still obscure. Above all, we can see that the 3 isoforms of Fbxw7 are differentially regulated and cannot be categorized as one protein.

### Biochemical Function as Ubiquitin Ligases to Degradate Target Proteins

**FIGURE 1:**
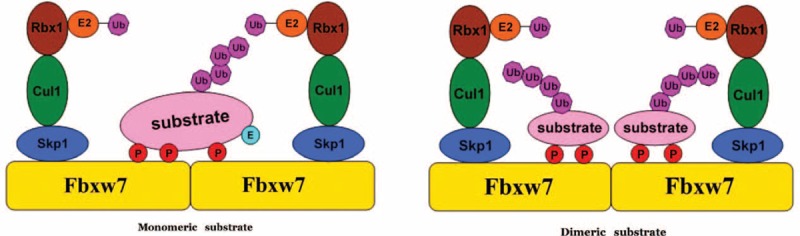
Schematic illustration of the SCF-type of E3 ubiquitin ligase complex (modified from ref.).^43^ The SCF (Skp1-Cullin 1-F-box) complex consists of 4 components: Skp1, Cul-1, Rbx1, along with the F-box protein family that play as substrate recognition components. Fbxw7 is a member of the F-box protein family, which recognizes the targeted substrates. High-affinity substrates have a consensus phosphopeptide motif termed CPDs that contain 2 phosphorylated residues (red “P”); lower-affinity CPDs contain a negatively charged amino acid (blue “E”) in place of the second phosphate. Fbxw7 also contains a conserved dimerization motif called the D domain, mediates Fbxw7 dimerization. CPD = Cdc4 phospho-degron, SCF = SKP1-CUL1-F-box.

The ubiquitin-proteasome pathway plays a vital role in many cellular functions by determining the abundance of cellular proteins, including short-lived, regulatory, and misfolded/denatured proteins. Dysregulation of the proteolytic system would result in uncontrolled proliferation, genomic instability, and leading to tumorigenesis. Ubiquitylation needs the concerted action of ubiquitin-activating enzymes (E1s), ubiquitin-conjugating enzymes (E2s), and ubiquitin ligases (E3s). The E2s have been emerged as key mediators of chain assembly, and the E3s mainly decide the substrate specificity. The cell cycle, in particular, is primarily controlled by 2 ubiquitin ligases, SCF and anaphase promoting complex/cyclosome, and perturbation of their function can result in tumorigenesis. SCF ubiquitin ligases compose of an invariable core complex of Cul1, Skp1, and Rbx1, along with the F-box protein family that play as substrate recognition components (Figure 1). Fbxw7 is a member of the F-box protein family, which is the component of an SCF E3 ubiquitin ligase. It contributes to the ubiquitin-mediated degradation of c-Myb,^6^ Mediator 13,^7^ Kruppel-like factor 2,^8^ Kruppel-like factor 5 (KLF5),^9^ granulocyte colony stimulating factor receptor,^10^ eya1,^11^ BCL-3,^12^ neurofibromatosis type 1,^13^ nuclear factor E2-related factor 1,^14^ p100/nuclear factor-κB2 (NF-κB2),^15,16^ GATA3,^17^ JunB,^18^ Myeloid cell leukemia-1 (Mcl-1),^19,20^ c-Myc,^21–27^ CyclinE,^21–27^ CDK2,^16^ Hes-1,^16^ CyclinD1,^16^ sterol regulatory element binding protein (SREBP),^28^ c-Jun,^23,29^ Hypoxia inducible factor-1α (HIF-1α),^30^ Notch1,^23,31,32^ DEK,^23^ Enolase 1 (ENO1),^33^ Yes-associated protein (YAP),^34^ mammalian target of rapamycin (mTOR),^35,36^ Ki-67,^24,37^ TOP2A,^20^ coiled-coil-domain containing 6,^38^ Aurora kinase A (Aurora-A),^26,37,39^ Notch4,^37^ proliferation cell nuclear antigen, and^37^ MYCN.^40^ They all function as cell-cycle promoters or oncogenic regulators of proliferation, growth, and apoptosis. The loss of Fbxw7 results in accumulation of its substrates, which leads to oncogenesis. However, little is known about which accumulating substrate is most related to human tumorigenesis in Fbxw7 deficiency cancer cells.

The 3rd, 4th repeat domain of the WD40 of Fbxw7 contains a highly conserved arginine residues (R465, R479, and R505), which bind with high affinity to Cdc4 phospho-degron (CPD), a consensus phosphopeptide motif^41^ (Figure 1). CPD is the common phosphorylation motif of most Fbxw7 substrates. When glutamate or phosphorylation offers a negative charge, the serine or threonine in the “+4” position of CPD can be phosphorylated. The phosphorylation of CPD is very important for recognition and subsequent degradation by Fbxw7. In some cases, the substrates of Fbxw7, including c-Myc, Cyclin E, c-Jun, SREBP, Notch, and c-Myb, have mutations within their CPDs, resulting in escaping from Fbxw7-mediated degradation.^42^

Fbxw7 also contains a conserved dimerization motif called the D domain (Figure 1), whose protomer consists of 3 α helices which self-associate in a right-handed superhelical manner to form a parallel dimer. The D domain mediates dimerization in a homotypic fashion, and the dimerization of the SCF complex facilitates ubiquitin conjugation but not substrate recognition. The D domain may accommodate considerable interaction specificity.^41^ For example, Sic 1, the target protein of Fbxw7 with weak affinity in yeast, has no CPD with high affinity, only carries several degradation determinants of low affinity, whose combination with Fbxw7 requires the help of Fbxw7 dimerization.^44^ Other substrates, which do not provide a negatively charged amino acid in the +4 position, or which cannot accommodate an extra phosphate in their phospho-degrons, may be absolutely dependent on Fbxw7 dimerization for their turnover.^45^

Glycogen synthase kinase 3 (GSK-3) is also the coregulator for turnover of some Fbxw7 substrates, which has the conserved phospho-epitope as the CPDs. It phosphorylates the central threonine or serine of the CPD in each substrate. GSK-3 has been found to phosphorylate many proteins playing crucial roles in a variety of cellular processes, such as cell proliferation, differentiation, and apoptosis. Study from Flugel et al^46^ indicated that phosphorylation of HIF-1α by GSK-3β recruited Fbxw7, which then promoted ubiquitylation of the HIF-1α protein. Pérez-Benavente and Farras^18^ demonstrated that GSK3β-mediated phosphorylation of JunB on a vital consensus phosphodegron that induced Fbxw7 E3-ligase recruitment and its degradation in late G2. Dysregulation of GSK3β-FBXW7-JUNB axis may be relevant in cancer.

Polo-like kinases perform important functions during mitosis, cytokinesis, and centriole duplication. Plk2 is involved in the reproduction of centrosomes and is activated in early G1 phase. However, little is known about the mechanisms underlying Plk2-induced centriole duplication. Cizmecioglu et al^47^ showed that Plk2 phosphorylated Fbxw7 on serine 176 and the 2 proteins (Polo-like kinases and Fbxw7) form a complex in vitro and in vivo, ultimately decreased the Fbxw7 protein stability, resulting in accumulation of cyclin E and increased potential for centriole reproduction. As we know, substrate phosphorylation is the main mechanism that ensures timely destruction of Fbxw7 substrates. Schulein et al^48^ showed that PI3K-dependent phosphorylation of Fbxw7 stimulated its ability to ubiquitinate and degrade its substrates, which controlled the balance between turnover of Fbxw7 and its substrates to fine-tune their activity. Another study showed that the novel PI3 kinase inhibitor NVP-BKM120 decreased Mcl-1 levels through facilitating its degradation in a GSK3/FBXW7-dependent mechanism, that contributed to induction of apoptosis and enhancement of trail-induced apoptosis in human lung cancer cells.^19^ In a study from Isobe et al,^49^ they showed that early region 1A, an oncogene product derived from adenovirus, interacted with Fbxw7 and attenuated the ubiquitylation of its target proteins in vivo. It may be helpful to explain the mechanism whereby adenovirus infection induced unregulated proliferation.

### Regulation of Fbxw7 activity

Regulation of kinase activity is important to maintain homeostasis as inappropriate activation or inhibition can always lead to cancer development. Fbxw7 is a tumor suppressor that controls the protein levels of many oncogenes. However, little is known about the regulation of Fbxw7 itself. There are some established mechanisms of regulation that can be applied to the regulation of Fbxw7: regulation of expression is at the transcription and protein level, and posttranslation modifications such as phosphorylation. But sometimes, the ways of regulation are different in 3 Fbxw7 isoforms.

#### p53

Fbxw7 is a p53-dependent tumor suppressor and its activation by p53 results in ubiquitination-mediated suppression of several oncoproteins. For example, Mao et al^50^ reported that Fbxw7 expression was upregulated when p53 expression was induced by radiation, and the baseline expression of Fbxw7 was suppressed in p53^−/−^ mice. Furthermore, p53-dependent loss of Fbxw7 led to genetic instability by mechanisms that involved the activation of Aurora-A, c-Jun, and Notch4, and they also found a p53-binding site that was consisted in a promoter region of the Fbxw7. In glioblastoma, Kimura et al^41^ found that Fbxw7β expression, but not the alternative isoform of this gene, Fbxw7a or Fbxw7γ, was activated in a p53-dependent manner in response to genotoxic stresses, such as UV irradiation and adriamycin treatment, suggesting that each isoform had a different functional role. In gastric cancers (GCs), the deletion of 1 copy of Fbxw7 and TP53 were discovered in 45.5% and 21.2% of gastric tumors, respectively, both the Fbxw7 and TP53 messenger RNA (mRNA) expressions were lower in tumors than in paired nonneoplastic specimens. And it was associated with a more invasive phenotype in GC cell lines.^51^ Previous studies had shown that, compared with inactivation of p53 or Fbxw7 alone, the simultaneous disruption of p53 and Fbxw7, two cell cycle checkpoint genes, led to poorer prognosis in clinical GC. This report showed that the status of Fbxw7 and p53 was vital for prognosis determination of GC patients.^52^ In human hepatocellular carcinoma (HCC), Fbxw7 protein expression was negatively correlated with mutant p53^21^ and could be activated by adenoviral delivery of p53. Perez-Losada et al^53^ showed that Fbxw7 was a p53-transcriptional target that controlled genomic instability and tumor development in epithelial tumors. Grim et al^54^ initially found that Fbxw7 and p53 cooperatively suppressed advanced and chromosomally unstable intestinal cancer. Li et al^55^ found that Fbxw7-mutated colorectal cancer cells exhibited aberrant expression of phosphorylated-p53 at Serine-15. Li et al^39^ discovered that p53 mutation led to increased expression of miR-25 and downregulation of Fbxw7, resulting in elevated levels of Aurora-A, which is critically important for the rapid proliferation and aggressive behavior of prostatic small cell neuroendocrine carcinoma. These reports strongly suggest that transcription of Fbxw7 is regulated by p53 activity and identifying additional transcriptional regulators that modulate Fbxw7 expression will be helpful in understanding the role of Fbxw7 in tumorigenesis.

#### MicroRNAs (miRNAs) Including miR-223, miR-25, miR-27a, miR-182, miR-503, miR-129-5p, and miR-92a

Recently, miRNAs have increasingly become recognized as one of the regulatory genes which can bind mRNA through sequence complementarities and cause inhibition of protein translation and/or degradation of mRNA. The miRNA complexes can act as oncogenes or tumor suppressors in the development of cancers, finally affect the progression of human tumors and the prognosis of the patients. Lately, multiple studies have identified several miRNAs which can regulate Fbxw7 expression. MiR-223 had been shown to have a significant adverse impact on the survival of oesophageal squamous cell carcinoma patients through repression of the function of Fbxw7.^56^ In addition, miR-223 played as an oncogene by inhibiting the expression of Fbxw7 in human GC^57^ and T cell acute lymphoblastic leukemia.^58^ Eto et al^59^ also revealed that the miR-223/Fbxw7 pathway regulated the sensitivity of a human epidermal growth factor receptor 2-positive GC cell line to trastuzumab through the modulation of apoptosis. Zhou et al^60^ discovered that miR-223 promoted the cisplatin resistance of GC cell via regulating cell cycle by targeting Fbxw7. Lu et al^61^ demonstrated that miRNA-25 had important roles in reprogramming mouse fibroblast cells to induced pluripotent stem cells by regulating some candidate gene targets including Fbxw7. Xiang et al^62^ found that miRNA-25 was significantly upregulated in nonsmall cell lung cancer (NSCLC) and promoted NSCLC cells proliferation and motility partially by targeting Fbxw7. Gong et al^63^ showed that miRNA-25 was overexpressed in primary tumor tissues of GC patients and promoted GC progression by directly downregulating Fbxw7 expression. In a study from Li et al,^64^ they showed that a sequential expression of miR-503 and miR-182 in benign adenoma cooperatively regulated Fbxw7, contributing to the malignant transformation of colon adenoma to adenocarcinoma. Lerner et al^65^ demonstrated that attenuation of Fbxw7 by miR-27a overexpression led to inappropriate cell cycle progression and DNA replication stress, in accordance with the dysregulation of cyclin E expression. Recently, over-expression of miR-129-5p was identified to upregulate Fbxw7 expression. However, the underlying mechanism is unclear.^66^ Zhou et al^67^ indicated that miR-92a was upregulated in cervical cancer and promoted cell proliferation and invasion by suppressing the expression level of Fbxw7. Therefore, the expression of Fbxw7 appears to be tightly regulated by several different miRNAs. However, so far, no studies have identified whether the regulation of miRNAs has much difference in Fbxw7α, Fbxw7β, and Fbxw7γ.

#### RBP-J-Interacting and Tubulin-Associated (RITA)

RITA is a novel RBP-J-interacting protein that downregulates Notch-mediated transcription. Recently, Wang et al^68^ initially explored its molecular mechanism in HCC, they demonstrated that noncancerous liver tissues exhibited increased RITA expression compared to HCC tissues. Moreover, RITA overexpression upregulated p53 and reduced cyclin E levels, and RITA levels were associated with cell proliferation and apoptosis. More recently, Wang et al^16^ found that RITA overexpression increased protein expression of Fbxw7 and p53 and downregulated the expression of cyclin E, cyclin D1, CDK2, Hes-1, and NF-κB p65. It indicated that RITA exerted tumor-suppressive effects in hepatocarcinogenesis.

#### SREBP2

The SREBP family of transcription factors controls lipid and cholesterol metabolism. These proteins are rapidly degraded by the ubiquitin-proteasome pathway. However, the signals and factors required for this are unknown. Some researches indicated that the phosphorylation-dependent degradation of the SREBP family of transcription factors was regulated by Fbxw7.^28^ But another study showed that SREBP2 regulated miR-182 by targeting Fbxw7, resulting in a feedback pathway to regulate SREBP transcriptional activity.^69,70^

#### NF-κB1

The NF-κB family proteins are well-known transcription factors in regulation of multiple gene transcription and cellular processes, including the control of cell survival, tumor invasion, stress response, and drug resistance.^71^ It has been well documented that the transcription factor NF-κB2 (p100/p52) is one of the candidate Fbxw7 substrates, and Fbxw7 promotes degradation of p100 in a GSK3β phosphorylation-dependent manner. It indicates that Fbxw7 may exert its tumor-suppressor function by regulating NF-kB activity.^72^ But recently, studies have shown that NF-kB signaling pathways regulate miR-223/FBXW7 axis in T-cell acute lymphoblastic leukemia.^73^ NF-κB1 inhibited Fbxw7 expression and then suppressed its target c-Myc protein degradation,^74^ indicating that NF-κB1 may be an upstream regulator of Fbxw7. The biological function of NF-κB1 and NF-κB2 are different, therefore, more studies and increased research efforts are needed to investigate the underlying mechanisms.

#### Regulation of Fbxw7 Activity by Other Factors

Emerging evidence has suggested that Fbxw7 could be regulated by some other factors such as prolyl isomerase 1 (Pin1), family with sequence similarity 83, and member D (FAM83D). For example, Min et al^75^ indicated that Pin1 might be an upstream regulator of Fbxw7. Indeed, Pin1 directly bound to Fbxw7 and disrupted Fbxw7 dimerization in a phosphorylation-dependent manner. Depletion of Pin1 upregulated the expression of Fbxw7 protein, subsequently decreased Mcl-1 abundance, leading to the inhibition of tumor cell proliferation and transformation, also enhancing Taxol sensitivity in cancer cells. Wang et al^29^ discovered that FAM83D downregulated Fbxw7 and upregulated Fbxw7 targets, such as c-Myc, c-Jun, and mTOR, then promoting cell proliferation and migration as well as invasion in breast cancer cells. Balamurugan et al^30^ reported that CCAAT/enhancer-binding protein-δ, an inflammatory response gene and candidate tumor suppressor, directly inhibited the expression of Fbxw7 and upregulated the substrates of Fbxw7, like mTOR and HIF-1α, enhancing the mTOR/Akt/S6K1 signaling and promoting breast tumor metastasis. Rocher-Ros et al^76^ indicated that presenilin played a novel role on epidermal growth and transformation by reciprocally regulating the Notch and EGFR signaling pathways through the ubiquitin ligase Fbxw7. Recently, Welcker et al^77^ found that the Epstein–Barr nuclear antigen 1-binding protein 2 (EBP2) behaved mostly like the Fbxw7 pseudo-substrate that directly bound to Fbxw7 and regulated Fbxw7's nucleolar localization. Study from Sancho et al^78^ demonstrated that the downstream Notch signaling effector Hes-5 directly repressed transcription of Fbxw7β. Moreover, they revealed that the NICD/Hes-5/FBXW7β positive feedback loop underlied Fbxw7 haploinsufficiency. Numb was found to be required for cell fate determination during the neuroblast division. Recently, Numb4, one of the predominant Numb isoform, has been shown to promote Fbxw7 ubiquitin ligase assembly and activation, leading to increased Notch degradation.^79^ Fbxw7-dependent substrate ubiquitination was antagonized by the Usp28 deubiquitinase. Schulein-Volk et al^80^ discovered that dual regulation of Fbxw7 activity by Usp28 (which was equivalently disrupted by loss or overexpression of Usp28) was a safeguard mechanism for maintaining physiological levels of proto-oncogenic Fbxw7 substrates. Mo et al^81^ suggested that activated serum-and glucocorticoid-inducible protein kinase1 inhibited the Notch1 signaling pathway via phosphorylation of Fbxw7 at serine 227.

### Role of Fbxw7 as Tumor Suppressor in Human Solid Tumors and Its Mechanisms for Inactivation

Tumor suppressors are defined as those in which loss of function leads to tumor formation. Fbxw7 is a tumor suppressor gene that is responsible for the degradation of several proto-oncogenes and its functional inactivation can dysregulate the cell division process, and potentially lead to tumorigenesis. Since then, an explosion of studies explicitly addressed the role of Fbxw7 in human tumors. Fbxw7 has been implicated in astrocytoma, and in cancers of the lung, breast, gastric, liver, pancreatic, cervix, and esophagus. Several mechanisms have been reported for the inactivation of Fbxw7 in the progress of human cancer including mutation, deletion, and hypermethylation, of which the Fbxw7 mutation is most common. A lot of effort has been concentrated on finding Fbxw7 mutation in various types of human cancers, which has shown that the overall point mutation frequency is 6% to 35% in human cancers with tissue specificity.^82^

#### Fbxw7 in Gastric Cancer

Lee et al^83^ reported that the Fbxw7 mutation rate in GC tissues was from 3.7% to 6% and did not differ in early or advanced GC, which might play a role in the prognosis of GC. Yokobori et al^52^ pointed out that Fbxw7 mRNA expression level in cancerous tissues was lower than that in noncancerous tissues. And patients with low Fbxw7 expression had a remarkable poorer prognosis than those with high Fbxw7 expression. Fbxw7 expression was associated with the progressive tumor size, lymph node metastasis, peritoneal dissemination, venous invasion, and clinical stage. Experiments had also shown that loss of heterozygosity of Fbxw7 occurred in 32% of early-onset gastric cancers, and the loss of Fbxw7 expression had a significant correlation with the upregulation of c-Myc.^22^ Furthermore, Li et al^84^ found that Fbxw7 induced tumor apoptosis and growth arrest and inhibited the epithelial-to-mesenchymal transition (EMT) in part by downregulating the RhoA signaling pathway in GC.

#### Fbxw7 in Colorectal Cancer

In the research of Rajagopalan et al,^85^ somatic mutations in the Fbxw7 gene were found in 22 of the 190 colorectal tumors patients. Most mutations occurred in exon 7th to 10th, and caused truncation of the protein at a position amino-terminal to the 4th WD40 domain, which interrupted the binding of Fbxw7 to its substrates. The inactivation of Fbxw7 would lead to an increase of cyclin E and cell morphological abnormalities. Babaei-Jadidi et al^23^ discovered that the expression level of Fbxw7 in colorectal cancer tissue was lower compared with the normal tissue, and the low expression of Fbxw7 was associated with the poor prognosis. Fbxw7 mutation enhanced expression of c-Myc and cyclin E proteins and upregulated cell proliferation. Fbxw7 mutation also resulted in accumulation of multiple substrates and occurred in impaired degradation of Notch, Jun, and DEK, which cooperatively led to carcinogenesis. Miyaki et al^86^ discovered that Fbxw7 mutations were 9% in hereditary nonpolyposis colorectal cancer, 9% in familial adenomatous polyposis carcinomas, and 10% in sporadic carcinomas. Frameshift mutations were observed in hereditary nonpolyposis colorectal cancer tumors, while single-base substitutions predominantly happened in familial adenomatous polyposis and sporadic tumors. Loss of heterozygosity at the chromosome 4q region in Fbxw7 gene was seldom detected in tumors. Almost 25% of patients with colorectal cancer had a reduced copy number of Fbxw7, and the incidence of the genetic alteration was concordantly increased with the progression of disease stage. Multivariate analysis revealed that Fbxw7 expression in colorectal cancer was an independent prognostic factor for 5 year survival following surgery. Fbxw7 may be a useful prognostic indicator in colorectal cancer. Zhan et al^33^ showed that Fbxw7 physically binds to ENO1 and targets ENO1 for ubiquitin-mediated degradation, and suppressed the ENO1-induced gene expression, lactate production, cell proliferation, and migration in colorectal cancer.

#### Fbxw7 in Hepatocellular Carcinoma

Since then, an explosion of studies explicitly addressed the role of Fbxw7 in HCC. Tu et al^34^ demonstrated that Fbxw7 expression was impaired in HCC tissues and loss of Fbxw7 expression was related to poor clinicopathological features including venous infiltration, large tumor size, high pathological grading and, advanced tumor node metastasis stage. Additionally, some researchers found that the low expression of Fbxw7 correlates with the tumor recurrence after hepatectomy in patients with HCC.^87^ Tu et al^21^ discovered that p53 contributed to hepatocarcinogenesis partially through the downregulation of Fbxw7 activity and the accumulation of c-Myc and cyclin E. Furthermore, Tu et al^34^ indicated that Fbxw7 was inversely associated with YAP protein expression, and it regulated YAP protein abundance by targeting YAP for ubiquitination and proteasomal degradation in HCC tissues. Yu et al^88^ reported that Fbxw7 increased chemosensitivity in HCC cell lines through suppression of EMT. These results indicate that Fbxw7 may serve as a prognostic marker and that c-Myc, cyclin E, YAP may be potential targets of Fbxw7 in HCC. Yang et al^89^ reported a crucial role of Fbxw7 in cholangiocarcinoma metastasis by regulating EMT.

#### Fbxw7 in Breast Cancer

In breast cancer, the somatic mutation rate of the Fbxw7 gene was found to be less than 1%, which was based on the catalogue of somatic mutations in cancer database. But deletions of chromosome 4q31 in which Fbxw7 located were found to be more than 30% of breast cancer cell lines and primary cancers.^90^ In addition, a study of breast cancer^91^ demonstrated another mechanism for inactivation of Fbxw7, namely promoter specific methylation. Methylation of Fbxw7 was related to favorable prognosis despite its association with poorly differentiated tumors. Wei et al^92^ found that the Fbxw7 expression level was significantly reduced in breast cancer compared to the normal breast tissues, and the lower level of Fbxw7 expression was associated with shorter disease-free survival, especially in patients with estrogen receptor-negative and basal subtype tumors. Silencing Fbxw7 enhanced expression of mTOR,^35^ c-Myc,^24^ Ki-67,^24^ cyclin E,^24^ and KLF5^93^ proteins and upregulated both cell proliferation and G1-S transition.

#### Fbxw7 in Lung Cancer

Villaruz et al^36^ firstly described the role of Fbxw7 mutation in NSCLC. They found the mTOR inhibitor temsirolimus was still effective in a patient with adenocarcinoma of the lung, who had previously progressed on multiple lines of systemic therapy. Furthermore, Fbxw7 upregulation significantly increased cisplatin cytotoxicity in NSCLC.^94^ Recently, Yokobori et al^20^ revealed that low Fbxw7 expression presented with more progressive cancer and shorter survival time than patients with high Fbxw7 expression. What is more, silenced Fbxw7 revealed enhanced MS-275 (a class I-specific histone deacetylase [HDAC] inhibitor) sensitivity and taxol resistance. Silencing Fbxw7 enhanced expression of Mcl-1^19,20^ and TOP2A.^20^ Zhao et al^38^ also showed that Fbxw7 interacted with and targeted coiled-coil-domain containing 6 for ubiquitin-mediated proteasomal degradation, but it could be impaired by Ataxia Telangiectasia Mutated during DNA damage response in lung cancer cells.

#### Fbxw7 in Glioma Malignancy

Glioma malignancy is the most common type of primary malignant brain tumor and may arise from a cell with neural stem-like properties. Until now, the silencing mechanisms for the Fbxw7 in gliomas were unknown, Gu et al^95^ suggested that mutation and methylation was not the major cause of the suppression of Fbxw7 in gliomas. Matsumoto et al^96^ implicated that Fbxw7 played an important role in the degradation of Notch family members, thus regarded as a pivotal regulator of “stemness” and neuronal-glial differentiation in neural stem cells. In the research of Kim et al,^25^ they proposed that p53 mutations led to gliomagenesis by both allowing the upregulation of c-Myc through downregulation of Fbxw7 and by protecting against c-Myc-induced apoptosis. Hagedorn et al^37^ found the expression level of Fbxw7 was significantly reduced in more than 80% grade IV glioma. Furthermore, in grade IV glioma biopsies, 2 targets of Fbxw7, Aurora-A and Notch4 were preferentially immunodetected. In human glioma cell lines, overexpression of Fbxw7 lost the expression of proliferation cell nuclear antigen,^37^ Ki-67,^37^ Cyclin E1,^26^ MYC,^26^ and AURKA.^26^ These observations suppose that Fbxw7 is a tumor suppressor in glioma malignancy, and interfering with Fbxw7 or its downstream targets would be a new therapeutic strategy.

#### Fbxw7 in Renal Malignancy

Williams et al^40^ identified Fbxw7 as a novel Wilms’ tumor gene, mutated or deleted in approximately 4% of tumors they examined, and they also found MYCN copy number gain in 9 of 104 (8.7%) cases, which was a target of Fbxw7-mediated ubiquitination and degradation. By using a rapid breakpoint cloning procedure in a case of renal cell cancer (RCC), Kuiper et al^97^ discovered that the Fbxw7 gene was disrupted by a constitutional t (3;4)(q21;q31). The analysis of the tumor tissue revealed the presence of some anomalies, concluding loss of the derivative chromosome 3. Therefore, disruption of the Fbxw7 gene might play a critical role in the development of human RCC. Fu et al^98^ found that the expression level of Fbxw7 in RCC tissues was highly related to its clinical pathologic grade and tumor node metastasis phase and was highly lower than in paracancerous normal tissues, and Fbxw7 overexpression suppressed RCC cell proliferation and induces apoptosis. These data suggested that Fbxw7 is a significant tumor suppressor gene in RCC.

#### Fbxw7 in Other Solid Tumors

In esophageal squamous cell carcinoma, cases with a loss of Fbxw7 copy number usually had low Fbxw7 expression and a poorer prognosis than those with no loss of copy number, and silencing Fbxw7 upregulated the expression of c-Myc, which played an important role in cell cycle regulation.^27^ In pancreatic cancer, Calhoun et al^99^ discovered that 6% of pancreatic adenocarcinomas overexpressed cyclin E, which was accompanied by a novel somatic homozygous mutation in Fbxw7. In addition, nuclear retention of Fbxw7 by specific inhibitors of nuclear export led to Notch1 degradation in pancreatic cancer.^31^ Furthermore, Ji et al^100^ found that Fbxw7 could be phosphorylated and destablized by KRAS mutation in pancreatic cancer. Aydin et al^32^ firstly provided evidence on Fbxw7 as a vital tumor suppressor mutated and inactivated in melanoma that resulted in sustained Notch1 activation and made Notch signaling inhibition as a promising therapeutic strategy in melanoma. Garcia-Dios et al^101^ found that mutations in Fbxw7 correlated with high endometrial cancer type, tumor grade, and lymph node status, which prompted that Fbxw7 played as a suppressor gene in endometrial carcinoma. In human nasopharyngeal carcinoma, Song ^102^ discovered that Fbxw7 increased drug sensitivity to cisplatin by downregulating the expression of multidrug resistance-associated protein. Li et al^103^ found that the expression of Fbxw7 in osteosarcoma (OS) cases were significantly lower than those in normal bone tissues, and the low expression of Fbxw7 was correlated with advanced clinical stage, high T classification, poor histological differentiation, and a worse 5-year survival of OS patients. Multivariate Cox regression analysis also indicated that Fbxw7 was an independent prognostic marker in OS.

## CONCLUSION

**FIGURE 2:**
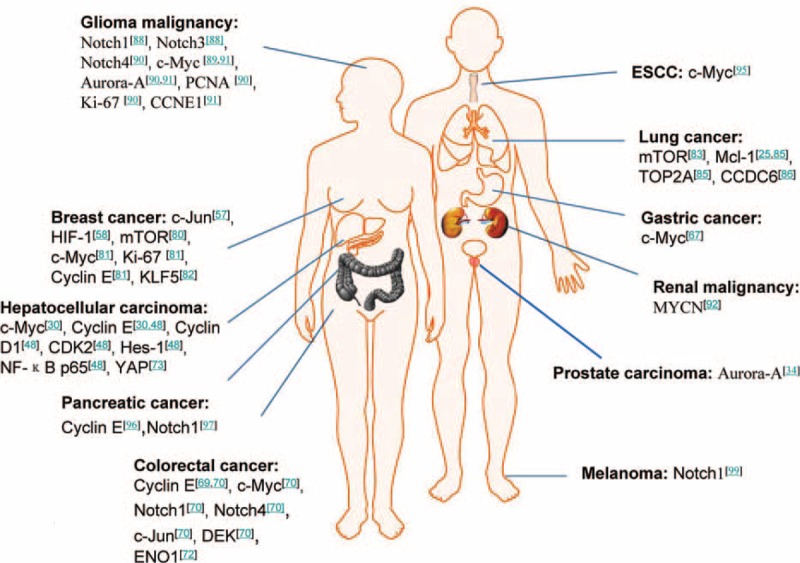
The downstream targets of F-box/WD repeat-containing protein 7 (Fbxw7) in different human solid tumor types. Fbxw7 coordinates the ubiquitin-dependent proteolysis of many key oncoproteins, and identifying critical Fbxw7 substrates are important for understanding tumorigenesis and discovering therapeutic targets. As shown above, in different human solid tumor types, the downstream targets of Fbxw7 are also different, which largely provide a new clue for the development of therapeutic targets in our fight against different cancer.

**FIGURE 3:**
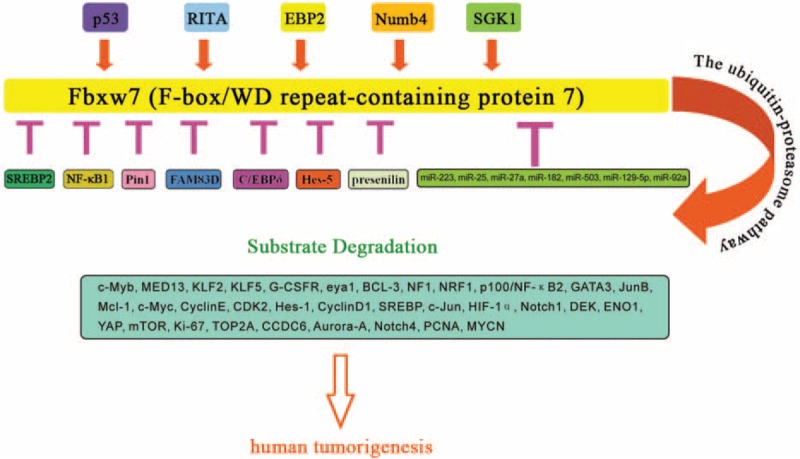
The upstream regulators of Fbxw7 and its major downstream targets that contributes to human tumorigenesis. Fbxw7 coordinates the ubiquitin-dependent proteolysis of several key oncoproteins, such as c-Myb,^6^ MED13,^7^ KLF2,^8^ KLF5,^9^ G-CSFR,^10^ eya1,^11^ BCL-3,^12^ NF1,^13^ NRF1,^14^ p100/NF-κB2,^15,16^ GATA3,^16^ JunB,^18^ Mcl-1,^19,20^ c-Myc,^21–27^ CyclinE,^21–27^ CDK2,^16^ Hes-1,^16^ CyclinD1,^16^ SREBP,^28^ c-Jun,^23,29^ HIF-1α,^30^ Notch1,^23,31,32^ DEK,^23^ ENO1,^33^ YAP,^34^ mTOR,^35,36^ Ki-67,^24,37^ TOP2A,^20^ CCDC6,^38^ Aurora-A,^26,37,39^ Notch4,^37^ PCNA,^37^ MYCN,^40^ and their function linked to defects in cell proliferation, differentiation, genetic instability, and ultimately tumorigenesis. What is more, several proteins such as p53, RITA, EBP2, Numb4, SGK1, SREBP2, NF-κB1, Pin1, FAM83D, C/EBPδ, Hes-5, presenilin, miR-223, miR-25, miR-27a, miR-182, miR-503, miR-129-5p, and miR-92a are found to regulate the expression of Fbxw7. Aurora-A = Aurora kinase A, CCDC6 = coiled-coil-domain containing 6, ENO1 = Enolase 1, Fbxw7 = F-box/WD repeat-containing protein 7, G-CSFR = Granulocyte colony stimulating factor receptor, HIF-1α = Hypoxia inducible factor-1α, KLF2 = Kruppel-like factor 2, KLF5 = Kruppel-like factor 5, Mcl-1 = Myeloid cell leukemia-1, MED13 = Mediator 13, mTOR = mammalian target of rapamycin, NF1 = Neurofibromatosis type 1, NF-κB2 = p100/Nuclear factor-κB2, NRF1 = Nuclear factor E2-related factor 1, PCNA = proliferation cell nuclear antigen, SREBP = sterol regulatory element binding protein, YAP = Yes-associated proteins.

This review has shed light on the magnitude of Fbxw7 within the human tumorigenesis and suggested that Fbxw7 is a vital tumor suppressor gene, which can be inactivated in the progress of human tumorigenesis through mutation, deletion, and hypermethylation, leading to an increase in several oncoproteins, including c-Jun, c-Myc, cyclin E, Notch1, mTOR, SREBP, c-Myb, Aurora-A, KLF5, Mcl-1, Neurofibromatosis type 1, Nuclear factor E2-related factor 1, ENO1, HIF-1α, and so on, most of which possess strong oncogenic roles. Furthermore, in different human solid tumor types, the downstream targets of Fbxw7 are also different (Figure 2). Fbxw7 contains 3 isoforms (Fbxw7α, Fbxw7β, and Fbxw7γ), and they are differently regulated in subtract recognition. Besides those, recently, accumulating evidence has shown that several molecules such as p53, miRNAs including miR-223, miR-25, miR-27a, miR-182, miR-503, and miR-129-5p, RITA, and FAM83D, as well as Pin1, CCAAT/enhancer-binding protein-δ, presenilin, SREBP2, NF-κB1, EBP2, Numb4 and serum-and glucocorticoid-inducible protein kinase1 could regulate Fbxw7 (Figure 3). Mutations of Fbxw7 are closely related to tumor progression and prognosis, and the detection of Fbxw7 mutations has potential clinical applications. In-depth study of Fbxw7 mutations will not only contribute to the diagnosis of cancer using Fbxw7 mutation as biomarkers, but also help in the development of targeted therapeutics for these mutations, which has positive implications for the prevention of cancer and individualized treatment.

## References

[1] Hartwell LH Mortimer RK Culotti J Genetic control of the cell division cycle in yeast: V. genetic analysis of cdc mutants. *Genetics* 1973; 74:267–286. 17248617 10.1093/genetics/74.2.267 PMC1212945

[2] Yang C Li S Wang M PTEN suppresses the oncogenic function of AIB1 through decreasing its protein stability via mechanism involving Fbw7 alpha. *Mol Cancer* 2013; 12:21. 23514585 10.1186/1476-4598-12-21 PMC3610140

[3] Strohmaier H Spruck CH Kaiser P Human F-box protein hCdc4 targets cyclin E for proteolysis and is mutated in a breast cancer cell line. *Nature* 2001; 413:316–322. 11565034 10.1038/35095076

[4] van Drogen F Sangfelt O Malyukova A Ubiquitylation of cyclin E requires the sequential function of SCF complexes containing distinct hCdc4 isoforms. *Molecular cell* 2006; 23:37–48. 16818231 10.1016/j.molcel.2006.05.020

[5] Sionov RV Netzer E Shaulian E Differential regulation of FBXW7 isoforms by various stress stimuli. *Cell Cycle* 2013; 12:3547–3554. 24091628 10.4161/cc.26591 PMC3906340

[6] Kitagawa K Hiramatsu Y Uchida C Fbw7 promotes ubiquitin-dependent degradation of c-Myb: involvement of GSK3-mediated phosphorylation of Thr-572 in mouse c-Myb. *Oncogene* 2009; 28:2393–2405. 19421138 10.1038/onc.2009.111

[7] Davis MA Larimore EA Fissel BM The SCF-Fbw7 ubiquitin ligase degrades MED13 and MED13L and regulates CDK8 module association with Mediator. *Genes Dev* 2013; 27:151–156. 23322298 10.1101/gad.207720.112 PMC3566307

[8] Wang R Wang Y Liu N FBW7 regulates endothelial functions by targeting KLF2 for ubiquitination and degradation. *Cell Res* 2013; 23:803–819. 23507969 10.1038/cr.2013.42 PMC3674386

[9] Luan Y Wang P FBW7-mediated ubiquitination and degradation of KLF5. *World J Biol Chem* 2014; 5:216–223. 24921010 10.4331/wjbc.v5.i2.216 PMC4050114

[10] Lochab S Pal P Kapoor I E3 ubiquitin ligase Fbw7 negatively regulates granulocytic differentiation by targeting G-CSFR for degradation. *Biochim Biophys Acta* 2013; 1833:2639–2652. 23820376 10.1016/j.bbamcr.2013.06.018

[11] Sun Y Li X The canonical wnt signal restricts the glycogen synthase kinase 3/fbw7-dependent ubiquitination and degradation of eya1 phosphatase. *Mol Cell Biol* 2014; 34:2409–2417. 24752894 10.1128/MCB.00104-14 PMC4054306

[12] Keutgens A Zhang X Shostak K BCL-3 degradation involves its polyubiquitination through a FBW7-independent pathway and its binding to the proteasome subunit PSMB1. *J Biol Chem* 2010; 285:25831–25840. 20558726 10.1074/jbc.M110.112128 PMC2919145

[13] Tan M Zhao Y Kim SJ SAG/RBX2/ROC2 E3 ubiquitin ligase is essential for vascular and neural development by targeting NF1 for degradation. *Dev Cell* 2011; 21:1062–1076. 22118770 10.1016/j.devcel.2011.09.014 PMC3241850

[14] Biswas M Phan D Watanabe M The Fbw7 tumor suppressor regulates nuclear factor E2-related factor 1 transcription factor turnover through proteasome-mediated proteolysis. *J Biol Chem* 2011; 286:39282–39289. 21953459 10.1074/jbc.M111.253807 PMC3234752

[15] Busino L Millman SE Scotto L Fbxw7alpha- and GSK3-mediated degradation of p100 is a pro-survival mechanism in multiple myeloma. *Nat Cell Biol* 2012; 14:375–385. 22388891 10.1038/ncb2463 PMC3339029

[16] Wang H Yang Z Liu C RBP-J-interacting and tubulin-associated protein induces apoptosis and cell cycle arrest in human hepatocellular carcinoma by activating the p53-Fbxw7 pathway. *Biochem Biophys Res Commun* 2014; 454:71–77. 25445601 10.1016/j.bbrc.2014.10.023

[17] Kitagawa K Shibata K Matsumoto A Fbw7 targets GATA3 through cyclin-dependent kinase 2-dependent proteolysis and contributes to regulation of T-cell development. *Mol Cell Biol* 2014; 34:2732–2744. 24820417 10.1128/MCB.01549-13 PMC4097664

[18] Perez-Benavente B Farras R Regulation of GSK3beta-FBXW7-JUNB axis. *Oncotarget* 2013; 4:956–957. 23918007 10.18632/oncotarget.1151 PMC3759673

[19] Ren H Zhao L Li Y The PI3 kinase inhibitor NVP-BKM120 induces GSK3/FBXW7-dependent Mcl-1 degradation, contributing to induction of apoptosis and enhancement of TRAIL-induced apoptosis. *Cancer Lett* 2013; 338:229–238. 23562472 10.1016/j.canlet.2013.03.032 PMC3750077

[20] Yokobori T Yokoyama Y Mogi A FBXW7 mediates chemotherapeutic sensitivity and prognosis in NSCLCs. *Mol Cancer Res* 2014; 12:32–37. 24165483 10.1158/1541-7786.MCR-13-0341

[21] Tu K Zheng X Zhou Z Recombinant human adenovirus-p53 injection induced apoptosis in hepatocellular carcinoma cell lines mediated by p53-Fbxw7 pathway, which controls c-Myc and cyclin E. *PloS One* 2013; 8:e68574. 23840897 10.1371/journal.pone.0068574 PMC3698167

[22] Milne AN Leguit R Corver WE Loss of CDC4/FBXW7 in gastric carcinoma. *Cell Oncol* 2010; 32:347–359. 20448329 10.3233/CLO-2010-523 PMC4619292

[23] Babaei-Jadidi R Li N Saadeddin A FBXW7 influences murine intestinal homeostasis and cancer, targeting Notch, Jun, and DEK for degradation. *J Exp Med* 2011; 208:295–312. 21282377 10.1084/jem.20100830 PMC3039859

[24] Ibusuki M Yamamoto Y Shinriki S Reduced expression of ubiquitin ligase FBXW7 mRNA is associated with poor prognosis in breast cancer patients. *Cancer Sci* 2011; 102:439–445. 21134077 10.1111/j.1349-7006.2010.01801.x

[25] Kim HS Woolard K Lai C Gliomagenesis arising from Pten- and Ink4a/Arf-deficient neural progenitor cells is mediated by the p53-Fbxw7/Cdc4 pathway, which controls c-Myc. *Cancer Res* 2012; 72:6065–6075. 22986743 10.1158/0008-5472.CAN-12-2594

[26] Gu Z Inomata K Ishizawa K The FBXW7 beta-form is suppressed in human glioma cells. *Biochem Biophys Res Commun* 2007; 354:992–998. 17274947 10.1016/j.bbrc.2007.01.080

[27] Yokobori T Mimori K Iwatsuki M Copy number loss of FBXW7 is related to gene expression and poor prognosis in esophageal squamous cell carcinoma. *Int J Oncol* 2012; 41:253–259. 22576686 10.3892/ijo.2012.1436

[28] Tu K Zheng X Yin G Evaluation of Fbxw7 expression and its correlation with expression of SREBP-1 in a mouse model of NAFLD. *Mol Med Rep* 2012; 6:525–530. 22710480 10.3892/mmr.2012.953

[29] Wang Z Liu Y Zhang P FAM83D promotes cell proliferation and motility by downregulating tumor suppressor gene FBXW7. *Oncotarget* 2013; 4:2476–2486. 24344117 10.18632/oncotarget.1581 PMC3926842

[30] Balamurugan K Wang JM Tsai HH The tumour suppressor C/EBPdelta inhibits FBXW7 expression and promotes mammary tumour metastasis. *EMBO J* 2010; 29:4106–4117. 21076392 10.1038/emboj.2010.280 PMC3018791

[31] Gao J Azmi AS Aboukameel A Nuclear retention of Fbw7 by specific inhibitors of nuclear export leads to Notch1 degradation in pancreatic cancer. *Oncotarget* 2014; 5:3444–3454. 24899509 10.18632/oncotarget.1813 PMC4116494

[32] Aydin IT Melamed RD Adams SJ FBXW7 mutations in melanoma and a new therapeutic paradigm. *J Natl Cancer Inst* 2014; 106:dju107. 24838835 10.1093/jnci/dju107 PMC4081626

[33] Zhan P Wang Y Zhao S FBXW7 negatively regulates ENO1 expression and function in colorectal cancer. *Lab Invest* 2015; 95:995–1004. 26097998 10.1038/labinvest.2015.71 PMC4552619

[34] Tu K Yang W Li C Fbxw7 is an independent prognostic marker and induces apoptosis and growth arrest by regulating YAP abundance in hepatocellular carcinoma. *Mol Cancer* 2014; 13:110. 24884509 10.1186/1476-4598-13-110 PMC4035898

[35] Mao JH Kim IJ Wu D FBXW7 targets mTOR for degradation and cooperates with PTEN in tumor suppression. *Science* 2008; 321:1499–1502. 18787170 10.1126/science.1162981 PMC2849753

[36] Villaruz LC Socinski MA Temsirolimus therapy in a patient with lung adenocarcinoma harboring an FBXW7 mutation. *Lung Cancer* 2014; 83:300–301. 24360397 10.1016/j.lungcan.2013.11.018 PMC4836187

[37] Hagedorn M Delugin M Abraldes I FBXW7/hCDC4 controls glioma cell proliferation in vitro and is a prognostic marker for survival in glioblastoma patients. *Cell Div* 2007; 2:9. 17326833 10.1186/1747-1028-2-9 PMC1819378

[38] Zhao J Tang J Men W FBXW7-mediated degradation of CCDC6 is impaired by ATM during DNA damage response in lung cancer cells. *FEBS Lett* 2012; 586:4257–4263. 23108047 10.1016/j.febslet.2012.10.029

[39] Li Z Sun Y Chen X p53 Mutation Directs AURKA Overexpression via miR-25 and FBXW7 in prostatic small cell neuroendocrine carcinoma. *Mol Cancer Res* 2015; 13:584–591. 25512615 10.1158/1541-7786.MCR-14-0277-T PMC4369163

[40] Williams RD Al-Saadi R Chagtai T Subtype-specific FBXW7 mutation and MYCN copy number gain in Wilms’ tumor. *Clin Cancer Res* 2010; 16:2036–2045. 20332316 10.1158/1078-0432.CCR-09-2890 PMC5122447

[41] Kimura T Gotoh M Nakamura Y hCDC4b, a regulator of cyclin E, as a direct transcriptional target of p53. *Cancer Sci* 2003; 94:431–436. 12824889 10.1111/j.1349-7006.2003.tb01460.x PMC11160198

[42] Tang X Orlicky S Lin Z Suprafacial orientation of the SCFCdc4 dimer accommodates multiple geometries for substrate ubiquitination. *Cell* 2007; 129:1165–1176. 17574027 10.1016/j.cell.2007.04.042

[43] Davis Ryan J Markus Welcker Clurman1 Bruce E Tumor suppression by the Fbw7 ubiquitin ligase: mechanisms and opportunities. *Cancer Cell* 2014; 26:455–464. 25314076 10.1016/j.ccell.2014.09.013 PMC4227608

[44] Orlicky S Tang X Willems A Structural basis for phosphodependent substrate selection and orientation by the SCFCdc4 ubiquitin ligase. *Cell* 2003; 112:243–256. 12553912 10.1016/s0092-8674(03)00034-5

[45] Welcker M Clurman BE Fbw7/hCDC4 dimerization regulates its substrate interactions. *Cell Div* 2007; 2:7. 17298674 10.1186/1747-1028-2-7 PMC1802738

[46] Flugel D Gorlach A Kietzmann T GSK-3beta regulates cell growth, migration, and angiogenesis via Fbw7 and USP28-dependent degradation of HIF-1alpha. *Blood* 2012; 119:1292–1301. 22144179 10.1182/blood-2011-08-375014 PMC3352078

[47] Cizmecioglu O Krause A Bahtz R Plk2 regulates centriole duplication through phosphorylation-mediated degradation of Fbxw7 (human Cdc4). *J Cell Sci* 2012; 125:981–992. 22399798 10.1242/jcs.095075

[48] Schulein C Eilers M Popov N PI3K-dependent phosphorylation of Fbw7 modulates substrate degradation and activity. *FEBS Lett* 2011; 585:2151–2157. 21620836 10.1016/j.febslet.2011.05.036

[49] Isobe T Hattori T Kitagawa K Adenovirus E1A inhibits SCF (Fbw7) ubiquitin ligase. *J Biol Chem* 2009; 284:27766–27779. 19679664 10.1074/jbc.M109.006809 PMC2788827

[50] Mao JH Perez-Losada J Wu D Fbxw7/Cdc4 is a p53-dependent, haploinsufficient tumour suppressor gene. *Nature* 2004; 432:775–779. 15592418 10.1038/nature03155

[51] Calcagno DQ Freitas VM Leal MF MYC, FBXW7 and TP53 copy number variation and expression in gastric cancer. *BMC Gastroenterol* 2013; 13:141. 24053468 10.1186/1471-230X-13-141 PMC3851138

[52] Yokobori T Mimori K Iwatsuki M p53-Altered FBXW7 expression determines poor prognosis in gastric cancer cases. *Cancer Res* 2009; 69:3788–3794. 19366810 10.1158/0008-5472.CAN-08-2846

[53] Perez-Losada J Mao JH Balmain A Control of genomic instability and epithelial tumor development by the p53-Fbxw7/Cdc4 pathway. *Cancer Res* 2005; 65:6488–6492. 16061623 10.1158/0008-5472.CAN-05-1294

[54] Grim JE Knoblaugh SE Guthrie KA Fbw7 and p53 cooperatively suppress advanced and chromosomally unstable intestinal cancer. *Mol Cell Biol* 2012; 32:2160–2167. 22473991 10.1128/MCB.00305-12 PMC3372235

[55] Li N Lorenzi F Kalakouti E FBXW7-mutated colorectal cancer cells exhibit aberrant expression of phosphorylated-p53 at Serine-15. *Oncotarget* 2015; 6:9240–9256. 25860929 10.18632/oncotarget.3284 PMC4496214

[56] Kurashige J Watanabe M Iwatsuki M Overexpression of microRNA-223 regulates the ubiquitin ligase FBXW7 in oesophageal squamous cell carcinoma. *Br J Cancer* 2012; 106:182–188. 22108521 10.1038/bjc.2011.509 PMC3251856

[57] Li J Guo Y Liang X MicroRNA-223 functions as an oncogene in human gastric cancer by targeting FBXW7/hCdc4. *J Cancer Res Clin Oncol* 2012; 138:763–774. 22270966 10.1007/s00432-012-1154-x PMC11824240

[58] Mansour MR Sanda T Lawton LN The TAL1 complex targets the FBXW7 tumor suppressor by activating miR-223 in human T cell acute lymphoblastic leukemia. *J Exp Med* 2013; 210:1545–1557. 23857984 10.1084/jem.20122516 PMC3727321

[59] Eto K Iwatsuki M Watanabe M The sensitivity of gastric cancer to trastuzumab is regulated by the miR-223/FBXW7 pathway. *Int J Cancer* 2015; 136:1537–1545. 25159729 10.1002/ijc.29168

[60] Zhou X Jin W Jia H MiR-223 promotes the cisplatin resistance of human gastric cancer cells via regulating cell cycle by targeting FBXW7. *J Exp Clin Cancer Res* 2015; 34:28. 25888377 10.1186/s13046-015-0145-6 PMC4387683

[61] Lu D Davis MP Abreu-Goodger C MiR-25 regulates Wwp2 and Fbxw7 and promotes reprogramming of mouse fibroblast cells to iPSCs. *PloS One* 2012; 7:e40938. 22912667 10.1371/journal.pone.0040938 PMC3422229

[62] Xiang J Hang JB Che JM miR-25 is up-regulated in non-small cell lung cancer and promotes cell proliferation and motility by targeting FBXW7. *Int J Clin Exp Pathol* 2015; 8:9147–9153. 26464659 PMC4583891

[63] Gong J Cui Z Li L MicroRNA-25 promotes gastric cancer proliferation, invasion, and migration by directly targeting F-box and WD-40 Domain Protein 7, FBXW7. *Tumour Biol* 2015; 36:7831–7840. 25944166 10.1007/s13277-015-3510-3

[64] Li L Sarver AL Khatri R Sequential expression of miR-182 and miR-503 cooperatively targets FBXW7, contributing to the malignant transformation of colon adenoma to adenocarcinoma. *J Pathol* 2014; 234:488–501. 25269767 10.1002/path.4407

[65] Lerner M Lundgren J Akhoondi S MiRNA-27a controls FBW7/hCDC4-dependent cyclin E degradation and cell cycle progression. *Cell Cycle* 2011; 10:2172–2183. 21597324 10.4161/cc.10.13.16248

[66] Hasler R Jacobs G Till A Microbial pattern recognition causes distinct functional micro-RNA signatures in primary human monocytes. *PloS One* 2012; 7:e31151. 22363568 10.1371/journal.pone.0031151 PMC3281918

[67] Zhou C Shen L Mao L miR-92a is upregulated in cervical cancer and promotes cell proliferation and invasion by targeting FBXW7. *Biochem Biophys Res Commun* 2015; 458:63–69. 25623537 10.1016/j.bbrc.2015.01.066

[68] Wang H Chen G Wang H RITA inhibits growth of human hepatocellular carcinoma through induction of apoptosis. *Oncol Res* 2013; 20:437–445. 24308154 10.3727/096504013x13685487925059

[69] Esquejo RM Jeon TI Osborne TF Lipid-cell cycle nexus: SREBP regulates microRNAs targeting Fbxw7. *Cell Cycle* 2014; 13:339–340. 24335356 10.4161/cc.27509 PMC3956523

[70] Jeon TI Esquejo RM Roqueta-Rivera M An SREBP-responsive microRNA operon contributes to a regulatory loop for intracellular lipid homeostasis. *Cell Metab* 2013; 18:51–61. 23823476 10.1016/j.cmet.2013.06.010 PMC3740797

[71] Ben-Neriah Y Karin M Inflammation meets cancer, with NF-kappaB as the matchmaker. *Nat Immunol* 2011; 12:715–723. 21772280 10.1038/ni.2060

[72] Arabi A Ullah K Branca RM Proteomic screen reveals Fbw7 as a modulator of the NF-kappaB pathway. *Nat Commun* 2012; 3:976. 22864569 10.1038/ncomms1975 PMC4354031

[73] Kumar V Palermo R Talora C Notch and NF-kB signaling pathways regulate miR-223/FBXW7 axis in T-cell acute lymphoblastic leukemia. *Leukemia* 2014; 28:2324–2335. 24727676 10.1038/leu.2014.133

[74] Huang H Ma L Li J NF-kappaB1 inhibits c-Myc protein degradation through suppression of FBW7 expression. *Oncotarget* 2014; 5:493–505. 24457827 10.18632/oncotarget.1643 PMC3964224

[75] Min SH Lau AW Lee TH Negative regulation of the stability and tumor suppressor function of Fbw7 by the Pin1 prolyl isomerase. *Mol Cell* 2012; 46:771–783. 22608923 10.1016/j.molcel.2012.04.012 PMC3389221

[76] Rocher-Ros V Marco S Mao JH Presenilin modulates EGFR signaling and cell transformation by regulating the ubiquitin ligase Fbw7. *Oncogene* 2010; 29:2950–2961. 20208556 10.1038/onc.2010.57 PMC4162408

[77] Welcker M Larimore EA Frappier L Nucleolar targeting of the fbw7 ubiquitin ligase by a pseudosubstrate and glycogen synthase kinase 3. *Mol Cell Biol* 2011; 31:1214–1224. 21220517 10.1128/MCB.01347-10 PMC3067902

[78] Sancho R Blake SM Tendeng C Fbw7 repression by hes5 creates a feedback loop that modulates Notch-mediated intestinal and neural stem cell fate decisions. *PLoS Biol* 2013; 11:e1001586. 23776410 10.1371/journal.pbio.1001586 PMC3679002

[79] Jiang X Xing H Kim TM Numb regulates glioma stem cell fate and growth by altering epidermal growth factor receptor and Skp1-Cullin-F-box ubiquitin ligase activity. *Stem Cells* 2012; 30:1313–1326. 22553175 10.1002/stem.1120 PMC3963835

[80] Schulein-Volk C Wolf E Zhu J Dual regulation of fbw7 function and oncogenic transformation by usp28. *Cell Rep* 2014; 9:1099–1109. 25437563 10.1016/j.celrep.2014.09.057

[81] Mo JS Ann EJ Yoon JH Serum- and glucocorticoid-inducible kinase 1 (SGK1) controls Notch1 signaling by downregulation of protein stability through Fbw7 ubiquitin ligase. *J Cell Sci* 2011; 124:100–112. 21147854 10.1242/jcs.073924

[82] Akhoondi S Sun D von der Lehr N FBXW7/hCDC4 is a general tumor suppressor in human cancer. *Cancer Res* 2007; 67:9006–9012. 17909001 10.1158/0008-5472.CAN-07-1320

[83] Lee JW Soung YH Kim HJ Mutational analysis of the hCDC4 gene in gastric carcinomas. *Eur J Cancer* 2006; 42:2369–2373. 16824748 10.1016/j.ejca.2005.10.034

[84] Li H Wang Z Zhang W Fbxw7 regulates tumor apoptosis, growth arrest and the epithelial-to-mesenchymal transition in part through the RhoA signaling pathway in gastric cancer. *Cancer Lett* 2015. 10.1016/j.canlet.2015.10.006 26458995

[85] Rajagopalan H Jallepalli PV Rago C Inactivation of hCDC4 can cause chromosomal instability. *Nature* 2004; 428:77–81. 14999283 10.1038/nature02313

[86] Miyaki M Yamaguchi T Iijima T Somatic mutations of the CDC4 (FBXW7) gene in hereditary colorectal tumors. *Oncology* 2009; 76:430–434. 19420964 10.1159/000217811

[87] Imura S Tovuu LO Utsunomiya T The role of Fbxw7 expression in hepatocellular carcinoma and adjacent non-tumor liver tissue. *J Gastroenterol Hepatol* 2014; 29:1822–1829. 24731221 10.1111/jgh.12623

[88] Yu J Zhang W Gao F FBW7 increases chemosensitivity in hepatocellular carcinoma cells through suppression of epithelial-mesenchymal transition. *Hepatobiliary Pancreatic Dis Int* 2014; 13:184–191. 10.1016/s1499-3872(14)60029-1 24686546

[89] Yang H Lu X Liu Z FBXW7 suppresses epithelial-mesenchymal transition, stemness and metastatic potential of cholangiocarcinoma cells. *Oncotarget* 2015; 6:6310–6325. 25749036 10.18632/oncotarget.3355 PMC4467439

[90] Chin K DeVries S Fridlyand J Genomic and transcriptional aberrations linked to breast cancer pathophysiologies. *Cancer Cell* 2006; 10:529–541. 17157792 10.1016/j.ccr.2006.10.009

[91] Akhoondi S Lindstrom L Widschwendter M Inactivation of FBXW7/hCDC4-beta expression by promoter hypermethylation is associated with favorable prognosis in primary breast cancer. *Breast Cancer Res* 2010; 12:R105. 21122106 10.1186/bcr2788 PMC3046450

[92] Wei G Wang Y Zhang P Evaluating the prognostic significance of FBXW7 expression level in human breast cancer by a meta-analysis of transcriptional profiles. *J Cancer Sci* 2012; 4:299–305. 10.4172/1948-5956.1000158 PMC3480227 23105958

[93] Zhao D Zheng HQ Zhou Z The Fbw7 tumor suppressor targets KLF5 for ubiquitin-mediated degradation and suppresses breast cell proliferation. *Cancer Res* 2010; 70:4728–4738. 20484041 10.1158/0008-5472.CAN-10-0040

[94] Yu HG Wei W Xia LH FBW7 upregulation enhances cisplatin cytotoxicity in non- small cell lung cancer cells. *Asian Pac J Cancer Prev* 2013; 14:6321–6326. 24377525 10.7314/apjcp.2013.14.11.6321

[95] Gu Z Inomata K Mitsui H Promoter hypermethylation is not the major mechanism for inactivation of the FBXW7 beta-form in human gliomas. *Genes Genet Syst* 2008; 83:347–352. 18931460 10.1266/ggs.83.347

[96] Matsumoto A Onoyama I Sunabori T Fbxw7-dependent degradation of Notch is required for control of “stemness” and neuronal-glial differentiation in neural stem cells. *J Biol Chem* 2011; 286:13754–13764. 21349854 10.1074/jbc.M110.194936 PMC3075719

[97] Kuiper RP Vreede L Venkatachalam R The tumor suppressor gene FBXW7 is disrupted by a constitutional t(3;4)(q21;q31) in a patient with renal cell cancer. *Cancer Genet Cytogenet* 2009; 195:105–111. 19963109 10.1016/j.cancergencyto.2009.07.001

[98] Fu Y Lin Y Yang Z FBXW7 overexpression suppresses renal cancer cell proliferation and induces apoptosis. *Med Oncol* 2015; 32:215. 26163148 10.1007/s12032-015-0656-1

[99] Calhoun ES Jones JB Ashfaq R BRAF and FBXW7 (CDC4, FBW7, AGO, SEL10) mutations in distinct subsets of pancreatic cancer: potential therapeutic targets. *Am J Pathol* 2003; 163:1255–1260. 14507635 10.1016/S0002-9440(10)63485-2 PMC1868306

[100] Ji S Qin Y Shi S ERK kinase phosphorylates and destabilizes the tumor suppressor FBW7 in pancreatic cancer. *Cell Res* 2015; 25:561–573. 25753158 10.1038/cr.2015.30 PMC4423074

[101] Garcia-Dios DA Lambrechts D Coenegrachts L High-throughput interrogation of PIK3CA, PTEN, KRAS, FBXW7 and TP53 mutations in primary endometrial carcinoma. *Gynecol Oncol* 2013; 128:327–334. 23219661 10.1016/j.ygyno.2012.11.037

[102] Song Y Zhou X Bai W FBW7 increases drug sensitivity to cisplatin in human nasopharyngeal carcinoma by downregulating the expression of multidrug resistance-associated protein. *Tumour Biol* 2015; 36:4197–4202. 25586348 10.1007/s13277-015-3056-4

[103] Li Z Xiao J Hu K FBXW7 acts as an independent prognostic marker and inhibits tumor growth in human osteosarcoma. *Int J Mol Sci* 2015; 16:2294–2306. 25622249 10.3390/ijms16022294 PMC4346837

